# Dr. Duvard’s Case of Catalepsy with Transposition of the Senses

**Published:** 1842-08-27

**Authors:** 

**Affiliations:** Caen


*Case of Catalepsy with Transposition of the Senses.* By Dr. Duvard, Caen.*—Mademoiselle Melanie had enjoyed good health up to the age of twenty-one, when she began to suffer from dry cough, with pain in the chest and headache; in January, 1841, she was attacked by pleurisy of the right side, and since then has continued to suffer from pain in that region; the catamenia now decreased in quantity and were finally arrested.

In the month of July, 1841, I was first called on to visit the patient; she then exhibited all the signs of pleuritic effusion. After a variety of treatment, continued for several weeks, a seton was inserted in the patient's side, and she was compelled to have an enema—a remedy which she had previously refused to submit to. A few hours after the administration of the enema, she was siezed with a most violent attack of hysteria, which continued for several hours. The attacks of hysteria recurred with the same violence, for several successive days, and seemed to be excited by the ingestion of food, which the patient continued to eat with avidity, in spite of remonstrances.

Six days after the first attack of hysteria, the patient became suddenly dumb, and continued so for three days, being unable to articulate a single word; on the 4th day she recovered the power of speech, at the termination of a severe hysterical attack. The surprise, however, expressed by those about her, at hearing her speak, threw her into a fresh fit, which lasted for three hours, and ended in catalepsy: this was on the 30th of August, 1841. From this period the patient was seized every day with several attacks of catalepsy, alternating with hysteria, and lasting about half an hour.

During the cataleptic accesses there was complete insensibility of every part of the body; the limbs remained in the most fatiguing positions without stirring; the respiratory movements were imperceptible, and the pulsations of the heart, which could scarcely be felt, were from 60 to 70 in the minute. After a few days the cataleptic fits became longer, and lasted for several hours, being, however, occasionally interrupted for a minute or two, whenever the girl coughed. Sometimes she would turn round in her bed or sit up; at

* We have abridged this curious case from the last number of the Gazette Médicale de Paris, for the edification of mesmerisers and miracle-mongers.—Eds.

others, she would suddenly start up, without opening the eyes, and place herself on the edge of the bed, or on some piece of furniture in a most fatiguing posture; in this state would she remain until a fit of coughing came on, or until she was brought back to her bed. Although the eyes were constantly shut, she avoided every obstacle carefully, and seemed heedless of risks which would have alarmed any one in a normal state. On one occasion she left her bed during a fit of coughing, ran to a window and opened it; before any one could come to her assistance, she had one foot out of the window, but the cough suddenly ceased, she became cataleptic, and remained in the same position until some people came and placed her in bed.

When the fits of hysteria and catalepsy ceased, the patient recovered all her faculties, and merely complained of fatigue, and her ordinary pain in the side.

Five weeks after the first attack of catalepsy, Mdlle. Melanie fell several times into a state of natural somnambulism. She would get up without opening her eyes, walk about her room, arrange the furniture, and enter into conversation with those about her, often mentioning circumstances which she would have wished to conceal; after remaining in this state for several hours, she fell into a state of catalepsy, indicated by apparent suspension of the respiration and complete silence.

On the first of October, a few days after her first access of somnambulism, I found the patient in a state of catalepsy. Having placed my hand on the epigastric region, I noticed that her countenance became expressive of pain. I then placed my lips on the pit of her stomach, and asked her several questions; to my infinite astonishment she answered correctly, for although I had read most of the histories of this kind recorded in different works, I did not believe one of them. During this first examination I made numerous experiments, which led me to conclude that there was a transposition of the five senses to the pit of the stomach. On the evening of this day I made fresh experiments, during three hours, in the presence of numerous witnesses, who were not less surprised than myself. In a word, during two months I renewed the experiments daily, and often several times a day, making use of every precaution to avoid deception, and having numerous witnesses around me. I shall now relate the results of these experiments.

During the cataleptic state the muscles presented three different conditions: Sometimes they were all relaxed, and the limbs could be placed in any position, which they retained, however fatiguing the posture might be; at other times all the muscles were in a state of rigid contraction; at others, again, they were relaxed, and the limbs fell down when raised from the body.

There was no sensibility in any part of the body, except over the pit of the stomach, the palms of the hands, and soles of the feet. Thus we might pinch the skin or pierce it with pins, pull out the hair, tickle the nose, &c., without eliciting any sign of feeling. On the contrary, if the pit of the stomach, soles of the feet, or palms of the hands were touched, even with the point of a feather, the girl immediately withdrew the part touched, and her countenance indicated displeasure. When a Leyden jar was placed in communication with the parts just named, she had a violent commotion, or was suddenly awakened, but the jar might be discharged on any other part of the body without producing the slightest effect.

The ears appeared to be insensible to sound—the loudest noise did not attract her attention; but when a small bell was agitated over the sensitive parts, her countenance shewed that she heard the noise. If the lips were placed in contact with the sensitive parts, she heard everything that was said, although the voice was so low that it could not possibly reach her ears. Her answers were delivered in an exceedingly low tone, and, generally speaking, the person appointed to catch them would repeat them, without having heard the questions asked. It was not necessary to place the lips in contact with the sensitive parts; I often employed a long stick, an iron rod, &c., as a conductor between the mouth of the speaker and the patient's foot, and she heard perfectly well, although the persons placed between her head and the speaker could not distinguish a syllable of the question asked.

The patient never spoke, except when her limbs were in a state of relaxation; during the rigid cataleptic state the tongue and organs of speech were immovable.

The senses of taste and smell were not exercised by their natural organs, but were very acute in the sensitive parts. Thus, we filled the nose with assafœtida, or tobacco; placed bottles of æther, concentrated ammonia, &c., under the nose, without producing the least effect; but when a small portion of a sapid body was placed in contact with the sensitive parts, the patient distinguished it at once. Thus she recognized and named, one after another, the syrups of poppies, vinegar, gum, and capillaire, wine, water, orange-flower water, Seidlitz water, currant jelly, &c., although only one or two drops of each substance was placed on the palm of her hand.

When a few grains of snuff were placed on the sole of her foot, she sneezed at once, and thus easily distinguished common French snuff from English snuff.

Although the results of my first experiments induced me to think that the sense of vision was transposed as well as the other senses, subsequent trials showed that what I had regarded as vision was nothing more than the result of an exquisite sense of touch. When an object was placed on any of the sensitive points, and she was asked if she saw it, she answered "yes," and immediately named the object if she were acquainted with it, or if not, gave a correct description of the body. Thus she always detected a watch, when placed over the pit of the stomach, and never failed to tell whether it was made of gold or silver, was going or stopped. If asked the hour, she would answer pretty correctly as to the true time of day; but if the hands of the watch were designedly changed, she always failed to tell what time they marked. She could distinguish and name every kind of French coin placed in her hand, but not the name of the sovereign under whose reign they were struck; she could distinguish a bit of silk from a bit of cloth, but not their respective colours.

At the second sitting, she succeeded in spelling the word *commerce*, written in large letters, and placed on the pit of the stomach; this required considerable efforts, and she complained for a long time that the attempt produced great fatigue; in subsequent experiments, however, she was never able to distinguish any of the letters of the alphabet, when placed in contact with the sensitive parts. Whenever I asked her to point out the seat of her disease, and indicate to us the appropriate remedies, she refused—answering that such was my business, and not hers.

[The remainder of Dr. Duvard's case is occupied with a history of the treatment, which it is unnecessary to describe. He attempted to magnetise the patient; during the first three sittings she fell asleep, and remained so for several hours, but afterwards all attempts of the kind failed to produce any effects. The use of electricity seemed to be attended with more beneficial results than any other remedy; after the first day the fits of catalepsy and hysteria became less frequent and violent, and the patient returned, much improved, to her friends in the country.]—***Prov. Med. Journ.***

